# Supplementary material for: Mechanistic study of plastic monomers in gestational diabetes mellitus: A network toxicology and molecular docking approach
Source: PLoS One. 2025 Dec 29;20(12):e0339863. doi: 10.1371/journal.pone.0339863 (PMC12747327; doi:10.1371/journal.pone.0339863)
Supplement: Attachment — Submitted filename: Reviewers comments.pdf [file pone.0339863.s002.pdf]

Comments:

This work is a wonderful attempt to find the correlations between the induced PM- toxicity and its implications in GDM. However, there are some shortcomings, which when addressed can improve the quality of the manuscript.

1. The gene targets are treated as independent entities. While the authors have utilized the interaction databases, but they have disregarded gene-gene interaction that can modulate the GDM risk.
2. The study focuses on molecular docking results which focuses on active sites only, ignoring the allosteric modulation mechanisms that could alter protein functions. I would suggest the authors to include molecular dynamic simulations study, any wet lab work, or to include and explain the possible allosteric effects of the target protein they are reviewing about. Since there are only three proteins (STAT3, AKT1 and TP53), it would be interesting to add on such contents.
3. The construction of the paper is not up to the mark. For example, targets like STAT3 and AKT are labelled as "core targets" without demonstrating their relevance to GDM pathogenesis and its function is general metabolic pathways. It would be better if the authors first explain the functions of the target protein in GDM vs. metabolism.
4. The study would be more validating if the authors provided with negative controls to confirm specificity of interactions.
5. It would be great if the authors present the whole pathway, and the molecular targets predicted along with their interactions in the form of a flow diagram.
6. Moreover, the entire GDM pathway is presented in a simplified form without addressing the crosstalk with other GDM-related pathways (PPARγ, AMPK etc.). Simply put, in studies such as the present one, the discussion is about discussing the effects of the results obtained to bring out some hypothetical conclusive statement. It will increase the quality of the manuscript if the authors reframe the discussion section with this in mind.
7. I even find that some of the most widely used plastics such as vinyl chloride and polyethylene are missing, and no justification has been provided for this exclusion. This calls for a more rigorous toxicity profiling.
8. The paper is presented as a conclusive statement for potential biomarkers by relying solely on computational predictions and databases. No distinction is made between hypothesis generating results and validated mechanisms. To rectify this, I recommend the authors to provide literature references. For example:

   The current study framework is this: *Monomer A interacts with protein 1 (GDM-related protein)*

Modified framework: *Monomer A interacts with protein 1 (GDM- related protein). This protein has been found to induce the expression of {gene/protein}* (reference) *which is implicated in GDM.*

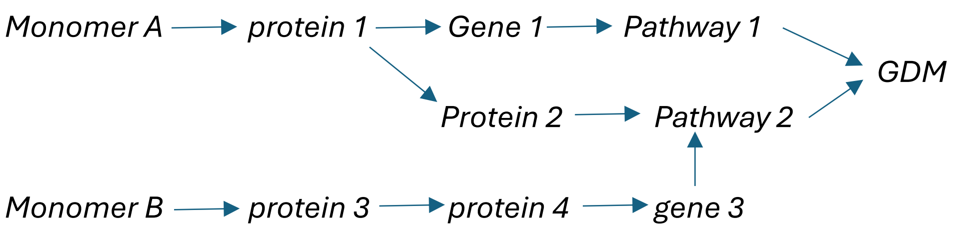

9. There is no discussion for correlation established between threshold concentrations of monomer toxicity and predicted binding affinities with real- life exposure scenarios. In real-life exposure, it is highly likely that monomer molecules of the polymer might be the only component which interacts with the cells, tissue of human body. The polymer structure as a whole is more likely to interact inducing toxicity.

Making these changes might lead to the increased practicality of the predicted biomarkers.
