# Supplementary material for: Mechanistic study of plastic monomers in gestational diabetes mellitus: A network toxicology and molecular docking approach
Source: PLoS One. 2025 Dec 29;20(12):e0339863. doi: 10.1371/journal.pone.0339863 (PMC12747327; doi:10.1371/journal.pone.0339863)
Supplement: Attachment — Submitted filename: Response to Reviewers .docx [file pone.0339863.s004.docx]

Dear Editor and Reviewers,

We would like to express our sincere gratitude to the Editor and Reviewers for their meticulous review and valuable insights on our manuscript (PONE-D-25-16737). Their constructive feedback has been instrumental in enhancing the overall quality and scientific rigor of our paper.

In response, we have revised the manuscript accordingly. Our point-by-point responses are provided below, and the revised manuscript file has been updated to reflect all changes.

**Comment 1: The gene targets are treated as independent entities. While the authors have utilized the interaction databases, but they have disregarded gene-gene interaction that can modulate the GDM risk.**

**Response:**

Thank you for your insightful comments and constructive feedback. We fully agree with your observation that gene targets do not function in isolation and that the interaction network among them plays a crucial role in modulating the risk of GDM. This perspective is central to our study, and we have placed significant emphasis on exploring these interactions. Below is a point-by-point response to your concerns:

In our research, we did not treat the gene targets as independent entities. On the contrary, we specifically highlighted that these core targets—STAT3, AKT1, and TP53—collectively mediate the pathogenesis of GDM through a complex protein-protein interaction (PPI) network. Our analytical approach included the following key steps, which directly address your concern regarding gene interactions:

1. Systematic Construction and Analysis of the PPI Network

We used the STRING database to construct a PPI network encompassing all potential targets. The STRING database is a authoritative resource for known and predicted protein interactions. Topological analysis and visualization were performed using Cytoscape, allowing us to identify hub genes within the network. It was through this network-based analysis—rather than evaluating individual genes in isolation—that we concluded STAT3, AKT1, and TP53 represent the most highly connected and functionally central nodes. This indicates that their pathogenic roles are achieved precisely through extensive interactions with other genes and proteins.

2. Detailed Discussion of the Functional Interactions Among Hub Genes

At the molecular level, this study confirmed that plastic monomers (PM) can bind to and interfere with the functions of TP53, AKT1, and MAPK1 proteins. TP53, AKT1, and MAPK1 have been reported to play critical roles in the pathogenesis and progression of GDM. The pathophysiological mechanisms of GDM involve impaired insulin secretion and signaling, and its progression is closely associated with multiple interrelated pathological processes, including inflammation, insulin resistance, and oxidative stress (Kung & Murphy, 2016; Mittal et al., 2025; Saucedo et al., 2023).

The transcription factor STAT3, as a downstream effector of various cytokines (including IL-6, IL-1β, and IL-33), promotes the development of GDM by inhibiting insulin secretion (Li et al., 2023). Through kinase-dependent activation (e.g., JNK, mTOR, PKCδ), nuclear translocation, and regulation of target gene transcription (such as SOCS family proteins), STAT3 significantly affects insulin sensitivity and promotes IRS1 ubiquitination and degradation, thereby disrupting insulin signal transduction (Den Hartogh et al., 2025; Salminen et al., 2021). Moreover, STAT3 expression is significantly elevated in the serum and placenta of GDM patients and is strongly associated with GDM occurrence (Li et al., 2023). Aberrant activation of STAT3 represents a core molecular event linking inflammation and insulin resistance in GDM.

AKT1, a key serine/threonine kinase in the PI3K/AKT signaling pathway, is involved in the regulation of glucose metabolism. Its dysfunction is closely associated with metabolic syndromes such as diabetes and dyslipidemia (Verma et al., 2023). Dysregulation of this pathway plays a central role in β-cell dysfunction and insulin resistance induced by abnormal lipid metabolism, involving mechanisms related to inflammation and oxidative stress (Kung & Murphy, 2016). Studies have shown that genes associated with the PI3K/AKT pathway (e.g., MMPs) are upregulated in placental tissues of GDM patients, and this pathway, together with Jak-STAT and mTOR signaling, participates in diabetes-related pathological processes in GDM (Huang et al., 2024; Zhang et al., 2024). Furthermore, multiple studies have demonstrated that modulating this pathway (e.g., via Apelin-13 activation or miR-22/372 inhibition) significantly improves glycolipid metabolism and insulin sensitivity in GDM, suggesting its therapeutic potential (Li et al., 2022; Zheng et al., 2021). Dysfunction of AKT1 directly contributes to metabolic dysregulation in GDM, serving as a key node connecting insulin signaling with glucose and lipid metabolism.

TP53 exacerbates the GDM phenotype by inducing insulin resistance and metabolic dysfunction (e.g., hepatic steatosis) (Derdak et al., 2011; Punja et al., 2021). It is closely associated with insulin resistance in adipose tissue and participates in the regulation of insulin sensitivity through the MAPK and NF-κB pathways (Geng et al., 2018; Minamino et al., 2009). Stressors such as hyperglycemia, free fatty acids, and inflammation can activate p53, thereby inducing β-cell apoptosis and impairing AKT-mediated cytoprotection, ultimately leading to compromised insulin secretion and glucose homeostasis (Kung & Murphy, 2016). Activation of TP53 promotes β-cell damage and insulin resistance in GDM pathogenesis, acting as a critical mediator between stress response and metabolic deterioration.

Additionally, studies have revealed that circ-DNMT1 promotes GDM progression by inducing p53 expression and activating the JAK/STAT pathway, while DUSP9 exacerbates insulin resistance and metabolic dysfunction by inhibiting the IRS1/PI3K/AKT pathway (Bao et al., 2022; Zhang & Jin, 2025). Conversely, SIRT1-mediated regulation of STAT3/SOCS3 and the targeting of the STAT3/FAM3A axis by miR-1299 modulate hepatic glucose homeostasis and insulin sensitivity in GDM at different levels(Chen et al., 2024; Wen et al., 2025).

These data converge to indicate that STAT3, AKT1, and TP53 do not act in isolation but form a functional synergistic axis. The STAT3–AKT1–TP53 core network integrates multiple pathological mechanisms—such as inflammation, oxidative stress, and insulin resistance—ultimately driving the onset and progression of GDM. This convergence of mechanisms offers potential targets for therapeutic intervention.

3. Pathway Enrichment Analysis Reveals Shared Biological Processes Underlying Interactions

Our KEGG pathway enrichment analysis (e.g., HIF-1 signaling, thyroid hormone signaling pathway) reflects common biological processes involving coordinated actions of multiple genes. These pathways exemplify how functionally related gene sets operate through interactions to carry out specific cellular functions. The significant enrichment of our core targets in these pathways strongly supports the notion that they collectively modulate GDM risk via network interactions.

In summary, the core of our methodological approach is network toxicology, which focuses on how chemical perturbations affect entire biomolecular networks rather than individual targets. Our workflow—from PPI network analysis to hub gene identification and functional interpretation—inherently emphasizes and directly analyzes gene interactions.

We greatly appreciate your comments, which have provided us with an opportunity to clarify the central premise of our study. We have already highlighted the importance of network interactions in the manuscript and have further strengthened these discussions in the revised version. We thank you for your thorough and insightful review

**Comment 2: The study focuses on molecular docking results which focuses on active sites only, ignoring the allosteric modulation mechanisms that could alter protein functions. I would suggest the authors to include molecular dynamic simulations study, any wet lab work, or to include and explain the possible allosteric effects of the target protein they are reviewing about. Since there are only three proteins (STAT3, AKT1 and TP53), it would be interesting to add on such contents.**

**Response:**

We thank the reviewer for their insightful and constructive comments. We agree with the valid point raised regarding the limitations of molecular docking, which primarily offers static, binding-site-oriented snapshots and cannot fully capture protein conformational dynamics or allosteric regulatory effects that may be induced by ligand binding. We fully acknowledge that molecular dynamics simulations and experimental validation represent the gold standard for confirming binding mechanisms and functional impacts.

Due to current limitations in experimental facilities and computational resources, we were unable to perform the suggested molecular dynamics simulations or wet-lab experiments within the scope of this study. We sincerely acknowledge this as a current limitation of our work. Should conditions permit in the future, we will prioritize the following studies to thoroughly validate and extend our findings:

1. Computational validation: Performing molecular dynamics simulations to analyze the stability, key conformational changes, and energy profiles of PM complexes with STAT3, AKT1, and TP53, thereby providing deeper insights into potential allosteric mechanisms.
2. Experimental validation: Designing experiments using surface plasmon resonance (SPR) to quantitatively determine binding affinity, along with employing Western blotting and other cellular assays to evaluate the actual effects of PM exposure on phosphorylation levels of key targets and related signaling pathways in cell models.

We believe that, despite current resource constraints, the integrated network pharmacology and molecular docking approach adopted in this study provides a novel and important hypothetical foundation regarding the molecular mechanisms linking PM exposure to GDM, while also identifying clear targets for subsequent validation. We note that in many high-quality studies within the field of network toxicology, similar methodology—primarily relying on molecular docking as a starting point for hypothesis generation—has been adopted during early-stage mechanistic exploration. For instance, Xie et al. in *Journal of Translational Medicine* used a comparable approach to investigate the molecular mechanisms linking artificial sweeteners to cancer risk (Xie et al., 2025), and Cheng et al. in *Ecotoxicology and Environmental Safety* applied such methods to explore how triclosan exposure might exacerbate ischemic stroke risk (Cheng et al., 2025). This strategy is well recognized as a common and feasible approach in preliminary mechanistic screening studies, as evidenced by its employment in numerous investigations within the field.

Once more, we express our gratitude for the reviewer’s valuable suggestions, which are greatly instructive for refining our research framework and planning future work.

**Comment 3: The construction of the paper is not up to the mark. For example, targets like STAT3 and AKT are labelled as “core targets” without demonstrating their relevance to GDM pathogenesis and its function is general metabolic pathways. It would be better if the authors first explain the functions of the target protein in GDM vs. metabolism.**

**Response:**

We sincerely thank the reviewer for their valuable comments. We fully agree that the identification of STAT3, AKT1, and TP53 as core targets requires thorough biological justification. It is essential to clarify their specific roles in the pathogenesis of GDM, rather than relying solely on computational ranking from network analysis. This will significantly strengthen the conclusiveness and persuasiveness of our findings.

Based on your suggestions, we have supplemented and reorganized the original content of the manuscript. The following explanations, which will be integrated into the Discussion section, provide a solid theoretical foundation—based on prior knowledge—for the determination of these core targets.

At the molecular level, this study confirmed that PM can bind to and interfere with the functions of TP53, AKT1, and MAPK1 proteins. TP53, AKT1, and MAPK1 have been reported to play critical roles in the pathogenesis and progression of GDM. The pathophysiological mechanisms of GDM involve impaired insulin secretion and signaling, and its progression is closely associated with multiple interrelated pathological processes, including inflammation, insulin resistance, and oxidative stress (Kung & Murphy, 2016; Mittal et al., 2025; Saucedo et al., 2023).

The transcription factor STAT3, as a downstream effector of various cytokines (including IL-6, IL-1β, and IL-33), promotes the development of GDM by inhibiting insulin secretion (Li et al., 2023). Through kinase-dependent activation (e.g., JNK, mTOR, PKCδ), nuclear translocation, and regulation of target gene transcription (such as SOCS family proteins), STAT3 significantly affects insulin sensitivity and promotes IRS1 ubiquitination and degradation, thereby disrupting insulin signal transduction (Den Hartogh et al., 2025; Salminen et al., 2021). Moreover, STAT3 expression is significantly elevated in the serum and placenta of GDM patients and is strongly associated with GDM occurrence (Li et al., 2023). Aberrant activation of STAT3 represents a core molecular event linking inflammation and insulin resistance in GDM.

AKT1, a key serine/threonine kinase in the PI3K/AKT signaling pathway, is involved in the regulation of glucose metabolism. Its dysfunction is closely associated with metabolic syndromes such as diabetes and dyslipidemia (Verma et al., 2023). Dysregulation of this pathway plays a central role in β-cell dysfunction and insulin resistance induced by abnormal lipid metabolism, involving mechanisms related to inflammation and oxidative stress (Kung & Murphy, 2016). Studies have shown that genes associated with the PI3K/AKT pathway (e.g., MMPs) are upregulated in placental tissues of GDM patients, and this pathway, together with Jak-STAT and mTOR signaling, participates in diabetes-related pathological processes in GDM (Huang et al., 2024; Zhang et al., 2024). Furthermore, multiple studies have demonstrated that modulating this pathway (e.g., via Apelin-13 activation or miR-22/372 inhibition) significantly improves glycolipid metabolism and insulin sensitivity in GDM, suggesting its therapeutic potential (Li et al., 2022; Zheng et al., 2021). Dysfunction of AKT1 directly contributes to metabolic dysregulation in GDM, serving as a key node connecting insulin signaling with glucose and lipid metabolism.

TP53 exacerbates the GDM phenotype by inducing insulin resistance and metabolic dysfunction (e.g., hepatic steatosis) (Derdak et al., 2011; Punja et al., 2021). It is closely associated with insulin resistance in adipose tissue and participates in the regulation of insulin sensitivity through the MAPK and NF-κB pathways (Geng et al., 2018; Minamino et al., 2009). Stressors such as hyperglycemia, free fatty acids, and inflammation can activate p53, thereby inducing β-cell apoptosis and impairing AKT-mediated cytoprotection, ultimately leading to compromised insulin secretion and glucose homeostasis (Kung & Murphy, 2016). Activation of TP53 promotes β-cell damage and insulin resistance in GDM pathogenesis, acting as a critical mediator between stress response and metabolic deterioration.

Additionally, studies have revealed that circ-DNMT1 promotes GDM progression by inducing p53 expression and activating the JAK/STAT pathway, while DUSP9 exacerbates insulin resistance and metabolic dysfunction by inhibiting the IRS1/PI3K/AKT pathway (Bao et al., 2022; Zhang & Jin, 2025). Conversely, SIRT1-mediated regulation of STAT3/SOCS3 and the targeting of the STAT3/FAM3A axis by miR-1299 modulate hepatic glucose homeostasis and insulin sensitivity in GDM at different levels(Chen et al., 2024; Wen et al., 2025). These findings collectively highlight that the STAT3–AKT1–TP53 core network integrates multiple pathological mechanisms in GDM, including inflammation, oxidative stress, and insulin resistance. The convergence of these mechanisms offers potential targets for therapeutic intervention.

We believe that these supplemental contents provide a solid, prior knowledge-based theoretical justification for designating these molecules as core targets, significantly strengthening the persuasiveness of our conclusions. This valuable suggestion has greatly improved our manuscript.

**Comment 4: The study would be more validating if the authors provided with negative controls to confirm specificity of interactions.**

**Response:**

We sincerely thank you for your valuable feedback. We fully agree with the importance of including negative controls, which is crucial for verifying the specificity of interactions.

First, we would like to clarify that the present study primarily employs computational biology approaches—including network toxicology and molecular docking—which operate under a research paradigm distinct from that of traditional wet-lab experiments. Within this framework, conducting experimental-level negative controls (e.g., using inactive proteins or unrelated compounds) falls beyond the current scope of this work.

Nevertheless, to ensure the reliability and specificity of our findings, we have incorporated rigorous validation strategies equivalent to the concept of negative controls at multiple key stages of the computational analysis, thereby minimizing potential false-positive results:

1. In Target Prediction

The analytical tools used inherently incorporate high built-in confidence thresholds. These methods and their validation have been published in high-quality, peer-reviewed academic journals, further supporting the tool’s reliability in prediction accuracy and computational rigor. This approach functions as a statistical filter, effectively excluding a large number of low-probability, random false-positive targets, thereby significantly enhancing the reliability of the target set used in subsequent analyses.

2. In Molecular Docking

We analyzed the binding modes and energy characteristics between the plastic monomer PM and the core targets. The results indicated low binding energy and stable binding conformations, suggesting specific binding rather than non-specific adsorption.

3. In Pathway Enrichment Analysis

We strictly applied adjusted p-values (FDR < 0.05) as the significance threshold. The high statistical significance confirms that the identified pathways were not randomly generated, thereby supporting the specificity of the compound–target–pathway interactions at the functional level.

This strategy—relying on computational rigor, statistical significance, and multiple internal validations to ensure specificity—is a common and widely accepted practice in the field of network toxicology. Similar approaches have been adopted in numerous studies published in high-impact journals such as *Ecotoxicology and Environmental Safety* and *Journal of Translational Medicine*. For example, He et al. utilized network toxicology and molecular docking to analyze the potential mechanisms of plasticizer-induced breast cancer (He et al., 2024), and Xie et al. applied similar methods to investigate the association between artificial sweeteners and cancer risk (Xie et al., 2025).

Again, we deeply appreciate your thorough review, which has greatly enhanced the rigor and completeness of this work. We fully acknowledge the ultimate importance of experimental validation and have explicitly stated in the Discussion section that subsequent wet-lab experiments will be conducted to further verify the current computational findings.

**Comment 5: It would be great if the authors present the whole pathway, and the molecular targets predicted along with their interactions in the form of a flow diagram.**

**Response:**

We sincerely thank the reviewer for their highly valuable suggestion. We fully agree that a summary flowchart would greatly enhance the clarity and persuasiveness of the study by visually illustrating the complete predictive pathway from PM exposure to GDM phenotype, as well as the interactions among various molecules.

The proposed flowchart accurately captures the core narrative we intended to convey. It has now been integrated into the conclusion section of the manuscript. We greatly appreciate the reviewer's input, as this flowchart effectively summarizes our research hypothesis and key findings.

**Comment 6: Moreover, the entire GDM pathway is presented in a simplified form without addressing the crosstalk with other GDM-related pathways (PPARγ, AMPK etc.). Simply put, in studies such as the present one, the discussion is about discussing the effects of the results obtained to bring out some hypothetical conclusive statement. It will increase the quality of the manuscript if the authors reframe the discussion section with this in mind.**

**Response:**

We sincerely thank the reviewer for their valuable comment. We fully agree that the original presentation of GDM-related pathways was oversimplified and did not adequately reflect the crosstalk with other pathways implicated in GDM, such as PPARγ and AMPK signaling. In response to this suggestion, we have thoroughly restructured and deepened the Discussion section. The revised version (or the content below) elaborates on the complex interactions between these pathways.

Based on existing research, the onset and progression of GDM involve complex interactions among multiple signaling pathways, with the PI3K/AKT/mTOR, PPARγ, and AMPK pathways playing key roles in regulating insulin sensitivity, glucolipid metabolism, and placental function. Multiple studies indicate a close functional relationship between the PI3K/AKT/mTOR and PPARγ pathways. For instance, pea protein hydrolysate alleviates GDM-related metabolic disorders and placental dysfunction by inhibiting the PI3K/AKT/mTOR/PPARγ signaling pathway (Yu et al., 2025). SIRT1 upregulates QKI5 to promote PPARγ expression and activate the PI3K/AKT pathway, thereby ameliorating GDM pathology (He et al., 2023)；Galacto-oligosaccharides significantly improve metabolic parameters in a GDM rat model by modulating the PPARs/PI3K/Akt pathway and gut microbiota (Wan et al., 2025). These studies collectively reveal that the PI3K/AKT/mTOR and PPARγ signaling pathways form a functionally synergistic regulatory axis in GDM, representing a critical target for intervening in metabolic disorders.

On the other hand, AMPK, as a cellular energy sensor, also holds a central position in the pathogenesis of GDM. AMPK regulates glucolipid metabolism, protein synthesis, mitochondrial biogenesis, and oxidative stress responses. Its dysfunction is closely associated with maternal hyperglycemia and abnormal fetal development. Studies have shown that AMPK activity is often suppressed in GDM patients, while mTOR signaling is excessively activated, thereby exacerbating metabolic abnormalities (Kumagai et al., 2018). AMPK and AKT often exhibit antagonistic effects under metabolic stress (Zhao et al., 2017), suggesting that they together constitute a finely tuned regulatory network in GDM. Furthermore, AMPK can crosstalk with accumulated p53 protein and HIF-1α signaling under hypoxic conditions (Chun & Kim, 2021), although the precise molecular mechanisms require further elucidation. Therefore, impaired AMPK function and its crosstalk with key nodes such as mTOR, AKT, and p53 are central to driving metabolic imbalance in GDM.

It is noteworthy that cross-talk exists between thyroid hormone receptors and various nuclear receptors, including PPAR, collectively participating in the regulation of cholesterol and glucose metabolism (Mullur et al., 2014). The competitive relationship between thyroid hormone receptors and PPARα may influence glucose-stimulated insulin secretion (Holness et al., 2008), hinting at the potential role of thyroid hormone status in GDM development through glucose metabolism. Additionally, research on SGLT-2 inhibitor drugs shows that they exert anti-tumor effects by modulating the AMPK/mTOR and HIF-1α pathways (Li et al., 2025), providing a new perspective on the overlap between pathways in metabolic diseases and cancer. This indicates that cross-talk between receptor families and the overlapping pathways across different disease areas offer new insights for a comprehensive understanding of the complex network mechanisms underlying GDM.

In summary, the molecular mechanism of GDM constitutes a multi-pathway, multi-level regulatory network involving key players such as PI3K/AKT/mTOR, PPARγ, AMPK, HIF-1α, and thyroid hormone signaling. The detailed mechanisms of their interactions require further in-depth investigation. We hypothesize that PM may interfere with the functions of key proteins like STAT3, AKT1, and TP53, impact the HIF-1 and thyroid hormone signaling pathways, drive inflammatory, oxidative stress, and insulin signal transduction responses, thereby disrupting the functional balance of critical metabolic pathways such as PPARγ and AMPK, and ultimately synergistically contribute to the core pathophysiological processes of GDM: insulin resistance and β-cell dysfunction.

**Comment 7: I even find that some of the most widely used plastics such as vinyl chloride and polyethylene are missing, and no justification has been provided for this exclusion. This calls for a more rigorous toxicity profiling.**

**Response:**

We sincerely thank the reviewer for their valuable comment. We fully agree that the inclusion of widely used PM such as vinyl chloride (from PVC) and ethylene (from PE) is crucial for a comprehensive assessment of the toxicity profile of plastic-related chemicals.

The decision to focus on monomers from ABS, PET, PC, PA66, PA6, and PMMA—rather than vinyl chloride (PVC) or ethylene (PE)—was primarily based on scientific and methodological considerations regarding predictability.

Firstly, from the perspective of prediction feasibility and database limitations, both vinyl chloride (C_₂_H_₃_Cl) and ethylene (C_₂_H_₄_) are extremely small and structurally simple molecules. Our reverse target prediction strategy heavily relies on computational tools such as PharmMapper and Swiss Target Prediction, which function by comparing query molecules against libraries of known active ligands based on structural or pharmacophore features. These simple molecules lack sufficient pharmacophore complexity to support high-confidence binding predictions with protein targets, making subsequent network pharmacology analysis unfeasible. This represents a fundamental methodological constraint that precluded their inclusion.

This research aims to establish a proof-of-concept framework to explore how PM with predictable complex interaction potential may perturb biological networks and contribute to GDM. We therefore prioritized monomers whose chemical structures are suitable for in silico target prediction to ensure analytical feasibility and reliability.

In direct response to this suggestion, we have added the following statement to the Discussion section to clarify the rationale for excluding ethylene and vinyl chloride: ***Due to their extremely simple molecular structures, ethylene and vinyl chloride lack the complex pharmacophore features required for reliable in silico target prediction. Structure-based similarity tools therefore failed to generate robust target predictions for these monomers. Consequently, these widely used PM were not included in the present analysis, which represents a limitation in the comprehensiveness of the compound toxicity profile assessed.***

Additionally, we now highlight in the Discussion that future investigations into such structurally simple monomers should employ effect-based testing strategies—such as high-throughput transcriptomics, functional screening, or focused in vitro toxicological assays—to circumvent the limitations of structure-based predictions.

We once again thank the reviewer for this insightful comment, which has significantly improved the comprehensiveness and scientific rigor of our work. Your suggestion also provides valuable direction for our future studies.

**Comment 8: The paper is presented as a conclusive statement for potential biomarkers by relying solely on computational predictions and databases. No distinction is made between hypothesis generating results and validated mechanisms. To rectify this, I recommend the authors to provide literature references.**

**Response:**

We sincerely thank you for this valuable and insightful comment. We fully agree that the terminology regarding biomarkers was inappropriately used in certain instances, and we apologize for any confusion caused by this lack of precision. We concur that the results derived from computational predictions and database analyses should be regarded as hypothesis-generating rather than conclusive mechanistic evidence. It is essential to construct a complete argumentative chain supported by literature evidence.

In response to your suggestion, we have made the following key revisions and additions to the manuscript:

1. Correction of definitive statements to emphasize their hypothetical nature

We have systematically reviewed the entire manuscript and modified all conclusive statements solely based on computational predictions regarding biomarker functions into more cautious, hypothetical formulations. For instance, we now state that PM may potentially interfere with the functions of key proteins such as STAT3, AKT1, and TP53, thereby influencing the HIF-1 and thyroid hormone signaling pathways, which could contribute to GDM. We have ensured that all predictions are explicitly described as preliminary, predictive, or inferential.

2. Comprehensive Supplementation with Literature Evidence to Strengthen the Argumentation

We have supplemented all key predictions with corresponding literature evidence to construct a logical chain from computational prediction → biologically plausible support → proposed hypothesis. Major additions include:

After describing the predicted binding of PMs to targets like TP53, AKT1, and STAT3, we have added discussions citing existing literature (e.g., Li et al., 2023; Huang et al., 2024) that report aberrant expression or dysfunction of these targets in placental tissues or serum from GDM patients, indicating that our predicted targets are highly relevant to known pathophysiology of the disease.

Regarding the enrichment of the HIF-1 and thyroid hormone signaling pathways, we have not only highlighted their computational identification but also cited multiple studies (e.g., Yin et al., 2024; Wu et al., 2023) demonstrating actual dysregulation of these pathways in clinical GDM samples, thereby providing independent real-world research support for our findings.

For pathways not enriched but critically important (e.g., PPARγ, AMPK), we have expanded the Discussion to include documented crosstalk between these pathways and our core network, contextualizing our predictions within a broader established biological framework.

3. Enhanced Limitation Statements and Future Directions in the Discussion

A new paragraph has been added in the Discussion section stating: ***The core findings of this study originate from computational biological models, and these results provide important hypothesis-generating insights into the potential mechanisms of PMs. However, these predictive outcomes still require functional validation through subsequent in vitro and in vivo experiments (e.g., molecular assays, gene knockdown/overexpression) to ultimately establish causal relationships.***

We deeply appreciate your rigorous and professional review, which has significantly improved the scientific accuracy and clarity of our manuscript. We believe that these revisions have strengthened the logical coherence and reliability of our arguments.

**Comment 9: There is no discussion for correlation established between threshold concentrations of monomer toxicity and predicted binding affinities with real- life exposure scenarios. In real-life exposure, it is highly likely that monomer molecules of the polymer might be the only component which interacts with the cells, tissue of human body. The polymer structure as a whole is more likely to interact inducing toxicity.**

**Response:**

We sincerely thank the reviewer for their valuable comments. The issues raised regarding the concentration thresholds of monomers in real exposure scenarios and the combined toxic effects of polymers are indeed critical in environmental health risk assessment.

Firstly, we would like to clarify that the primary objective of this study is to employ computational toxicology approaches—namely network toxicology and molecular docking—to systematically screen and predict the potential molecular mechanisms and key targets through which PMs may contribute to GDM. As the reviewer is aware, research in this field remains at an early exploratory stage, and there is a severe lack of human exposure concentration and toxicity threshold data specific to GDM that could reliably guide such computational analyses. Therefore, conducting a precise correlation analysis between concentration, binding affinity, and biological effect poses significant challenges at this time. The main contribution of this work lies in providing priority hypotheses and mechanistic clues for this emerging research direction, rather than delivering a quantitative risk assessment. Subsequent confirmatory studies, including in vitro and in vivo models, will rely on the directional guidance generated by this type of computational work.

Secondly, this research focuses on the toxicity mechanisms of free monomers, based on the core scientific hypothesis that plastic products release biologically active monomeric compounds during environmental degradation or metabolic processes in vivo. These free monomers represent one of the direct forms mediating biological effects. We fully agree with the reviewer that real-world exposure is highly complex, and that the combined effects of polymers (such as micro- and nanoplastics), their role as carriers of pollutants, and mixture (“cocktail”) effects are of great importance. However, the toxicity mechanisms of polymer particles (e.g., physical damage, oxidative stress, inflammatory responses) and the molecular initiation mechanisms of free monomers (e.g., specific protein interactions, signaling pathway disruptions) represent distinct scientific questions. The latter is the explicit focus of this work.

As already stated in the Discussion section: ***This study primarily focuses on the toxicity of free monomers and does not fully address the potential composite toxic effects that may arise from microplastics/nanoplastics in real exposure scenarios.” We acknowledge this as a limitation of the present work, but also emphasize that this focus is necessary to gain in-depth insight into a relatively overlooked mechanism—molecular initiation by monomers. While numerous excellent studies have addressed microplastic toxicology, the exploration of specific mechanisms of monomer molecules has received comparatively less attention.***

In summary, the reviewer’s comments have highlighted important directions for future research. The current study emphasizes mechanism discovery, while subsequent work will build upon these predictive mechanisms by integrating actual environmental concentration monitoring, physiologically based pharmacokinetic modeling, and experimental validation in complex exposure systems (including both monomers and microplastics), thereby progressively advancing toward the higher-level risk assessment that the reviewer rightly encourages. We once again express our gratitude for the reviewer’s insightful suggestions, which have significantly enhanced our understanding of the complexity of this research topic.

**We sincerely thank the reviewers for their time and effort in evaluating our manuscript and for providing such thorough and insightful comments. Your expertise and valuable suggestions have greatly guided us in substantially improving this work. We feel privileged to have benefited from your expertise and hope that the revised manuscript meets your approval. Once again, we deeply appreciate your generous support.**

**References**

Bao, D., Zhuang, C., Jiao, Y., & Yang, L. (2022). The possible involvement of circRNA DMNT1/p53/JAK/STAT in gestational diabetes mellitus and preeclampsia. *Cell Death Discov*, *8*(1), 121. <https://doi.org/10.1038/s41420-022-00913-w>

Chen, X., Tao, X., Wang, M., Cannon, R. D., Chen, B., Yu, X., Qi, H., Saffery, R., Baker, P. N., Zhou, X., Han, T. L., & Zhang, H. (2024). Circulating extracellular vesicle-derived miR-1299 disrupts hepatic glucose homeostasis by targeting the STAT3/FAM3A axis in gestational diabetes mellitus. *J Nanobiotechnology*, *22*(1), 509. <https://doi.org/10.1186/s12951-024-02766-0>

Cheng, F., Gao, H., Yan, B., Chen, F., & Lei, P. (2025). Triclosan exposure potentiates ischemic stroke risk: Multi-omics integration and molecular docking unveil neurotoxic mechanisms. *Ecotoxicol Environ Saf*, *302*, 118551. <https://doi.org/10.1016/j.ecoenv.2025.118551>

Chun, Y., & Kim, J. (2021). AMPK-mTOR Signaling and Cellular Adaptations in Hypoxia. *Int J Mol Sci*, *22*(18). <https://doi.org/10.3390/ijms22189765>

Den Hartogh, D., MacPherson, R., & Tsiani, E. (2025). Muscle cell palmitate-induced insulin resistance, JNK, IKK/NF-κB, and STAT3 activation are attenuated by carnosic and rosmarinic acid. *Appl Physiol Nutr Metab*, *50*, 1-14. <https://doi.org/10.1139/apnm-2024-0302>

Derdak, Z., Lang, C. H., Villegas, K. A., Tong, M., Mark, N. M., de la Monte, S. M., & Wands, J. R. (2011). Activation of p53 enhances apoptosis and insulin resistance in a rat model of alcoholic liver disease. *J Hepatol*, *54*(1), 164-172. <https://doi.org/10.1016/j.jhep.2010.08.007>

Geng, S., Zhu, W., Wang, S., Xie, C., Li, X., Wu, J., Li, Y., Chen, Y., Wang, X., Meng, Y., Zhang, Q., Chen, J., & Zhong, C. (2018). P53 modulates hepatic insulin sensitivity through NF-κB and p38/ERK MAPK pathways. *Biochem Biophys Res Commun*, *495*(3), 2139-2144. <https://doi.org/10.1016/j.bbrc.2017.12.085>

He, J., Fan, F., Li, J., Han, Y., Song, Y., Zhang, R., Xu, Y., Wu, H., & Fan, R. (2023). SIRT1 alleviates insulin resistance and respiratory distress in late preterm rats by activating QKI5-mediated PPARγ/PI3K/AKT pathway. *Cell Cycle*, *22*(21-22), 2449-2466. <https://doi.org/10.1080/15384101.2023.2297567>

He, N., Zhang, J., Liu, M., & Yin, L. (2024). Elucidating the mechanism of plasticizers inducing breast cancer through network toxicology and molecular docking analysis. *Ecotoxicology and Environmental Safety*, *284*, 116866. <https://doi.org/https://doi.org/10.1016/j.ecoenv.2024.116866>

Holness, M. J., Greenwood, G. K., Smith, N. D., & Sugden, M. C. (2008). PPARalpha activation and increased dietary lipid oppose thyroid hormone signaling and rescue impaired glucose-stimulated insulin secretion in hyperthyroidism. *Am J Physiol Endocrinol Metab*, *295*(6), E1380-1389. <https://doi.org/10.1152/ajpendo.90700.2008>

Huang, X., Yip, K., Nie, H., Chen, R., Wang, X., Wang, Y., Lin, W., & Li, R. (2024). ChIP-seq and RNA-seq Reveal the Involvement of Histone Lactylation Modification in Gestational Diabetes Mellitus. *J Proteome Res*, *23*(6), 1937-1947. <https://doi.org/10.1021/acs.jproteome.3c00727>

Kumagai, A., Itakura, A., Koya, D., & Kanasaki, K. (2018). AMP-Activated Protein (AMPK) in Pathophysiology of Pregnancy Complications. *Int J Mol Sci*, *19*(10). <https://doi.org/10.3390/ijms19103076>

Kung, C. P., & Murphy, M. E. (2016). The role of the p53 tumor suppressor in metabolism and diabetes. *J Endocrinol*, *231*(2), R61-r75. <https://doi.org/10.1530/joe-16-0324>

Li, J., Qiao, J., Zhang, S., Zhong, J., Ke, C., Lin, Y., Li, Y., Jin, Y., Yao, Y., Zhai, A., & Bi, C. (2025). Unveiling the anticancer potential of SGLT-2 inhibitors: mechanisms and prospects in clinical oncology-a narrative review. *Eur J Med Res*, *30*(1), 520. <https://doi.org/10.1186/s40001-025-02749-3>

Li, L., Zhang, Z., Li, H., Zhou, M., Li, F., Chu, C., Zhang, Y., Zhu, X., Ju, H., & Li, X. (2023). Research progress on the STAT signaling pathway in pregnancy and pregnancy-associated disorders. *Front Immunol*, *14*, 1331964. <https://doi.org/10.3389/fimmu.2023.1331964>

Li, W., Yuan, X., He, X., Yang, L., Wu, Y., Deng, X., Zeng, Y., Hu, K., & Tang, B. (2022). The downregulation of miR-22 and miR-372 may contribute to gestational diabetes mellitus through regulating glucose metabolism via the PI3K/AKT/GLUT4 pathway. *J Clin Lab Anal*, *36*(7), e24557. <https://doi.org/10.1002/jcla.24557>

Minamino, T., Orimo, M., Shimizu, I., Kunieda, T., Yokoyama, M., Ito, T., Nojima, A., Nabetani, A., Oike, Y., Matsubara, H., Ishikawa, F., & Komuro, I. (2009). A crucial role for adipose tissue p53 in the regulation of insulin resistance. *Nat Med*, *15*(9), 1082-1087. <https://doi.org/10.1038/nm.2014>

Mittal, R., Prasad, K., Lemos, J. R. N., Arevalo, G., & Hirani, K. (2025). Unveiling Gestational Diabetes: An Overview of Pathophysiology and Management. *Int J Mol Sci*, *26*(5). <https://doi.org/10.3390/ijms26052320>

Mullur, R., Liu, Y. Y., & Brent, G. A. (2014). Thyroid hormone regulation of metabolism. *Physiol Rev*, *94*(2), 355-382. <https://doi.org/10.1152/physrev.00030.2013>

Punja, H. K., Nanjappa, D. P., Babu, N., Kalladka, K., Shanti Priya Dias, B., Chakraborty, G., Rao, S. M., & Chakraborty, A. (2021). TP53 codon 72 polymorphism and type 2 diabetes: a case-control study in South Indian population. *Mol Biol Rep*, *48*(6), 5093-5097. <https://doi.org/10.1007/s11033-021-06505-8>

Salminen, A., Kaarniranta, K., & Kauppinen, A. (2021). Insulin/IGF-1 signaling promotes immunosuppression via the STAT3 pathway: impact on the aging process and age-related diseases. *Inflamm Res*, *70*(10-12), 1043-1061. <https://doi.org/10.1007/s00011-021-01498-3>

Saucedo, R., Ortega-Camarillo, C., Ferreira-Hermosillo, A., Díaz-Velázquez, M. F., Meixueiro-Calderón, C., & Valencia-Ortega, J. (2023). Role of Oxidative Stress and Inflammation in Gestational Diabetes Mellitus. *Antioxidants (Basel)*, *12*(10). <https://doi.org/10.3390/antiox12101812>

Verma, K., Jaiswal, R., Paliwal, S., Dwivedi, J., & Sharma, S. (2023). An insight into PI3k/Akt pathway and associated protein-protein interactions in metabolic syndrome: A recent update. *J Cell Biochem*, *124*(7), 923-942. <https://doi.org/10.1002/jcb.30433>

Wan, J., Zhu, J., Zeng, J., Zhou, H., & He, Y. (2025). Effect of Galactooligosaccharide on PPARs/PI3K/Akt Pathway and Gut Microbiota in High-Fat and High-Sugar Diet Combined with STZ-Induced GDM Rat Model. *Probiotics Antimicrob Proteins*, *17*(2), 888-902. <https://doi.org/10.1007/s12602-023-10186-z>

Wen, Y., Wang, J., Duan, M., Li, Q., & Ma, J. (2025). SIRT1-Mediated Regulation of STAT3/SOCS3 Inhibits Insulin Resistance in Trophoblast Cells and Improves Gestational Diabetes Mellitus. *Faseb j*, *39*(9), e70620. <https://doi.org/10.1096/fj.202500520R>

Xie, J., Zhu, Y., Yang, Z., Yu, Z., Yang, M., & Wang, Q. (2025). An integrative analysis reveals cancer risk associated with artificial sweeteners. *J Transl Med*, *23*(1), 32. <https://doi.org/10.1186/s12967-024-06047-0>

Yu, T., Shang, N., Chen, L., Lu, K., Xia, H., Yang, L., Wang, S., Sun, G., & Liao, W. (2025). The Regulatory Effects of Pea Protein Hydrolysate on Lipid Metabolism in Gestational Diabetic Mice. *Mol Nutr Food Res*, e70178. <https://doi.org/10.1002/mnfr.70178>

Zhang, X., & Jin, Y. (2025). DUSP9-mediated inhibition of IRS1/PI3K/AKT pathway contributes to insulin resistance and metabolic dysfunction in gestational diabetes mellitus. *Hum Immunol*, *86*(3), 111263. <https://doi.org/10.1016/j.humimm.2025.111263>

Zhang, Y., Liu, Y., Shi, Y., Bai, C., Wang, T., Ruan, F., & Hu, C. (2024). Upregulation of MMPs in placentas of patients with gestational diabetes mellitus: Involvement of the PI3K/Akt pathway. *Heliyon*, *10*(12), e32518. <https://doi.org/10.1016/j.heliyon.2024.e32518>

Zhao, Y., Hu, X., Liu, Y., Dong, S., Wen, Z., He, W., Zhang, S., Huang, Q., & Shi, M. (2017). ROS signaling under metabolic stress: cross-talk between AMPK and AKT pathway. *Mol Cancer*, *16*(1), 79. <https://doi.org/10.1186/s12943-017-0648-1>

Zheng, X. D., Huang, Y., & Li, H. (2021). Regulatory role of Apelin-13-mediated PI3K/AKT signaling pathway in the glucose and lipid metabolism of mouse with gestational diabetes mellitus. *Immunobiology*, *226*(5), 152135. <https://doi.org/10.1016/j.imbio.2021.152135>
